# Physical activity partly inhibits autonomic imbalance, immune dysfunction and quality of life induced by chronic shiftwork in policemen

**DOI:** 10.1113/EP094113

**Published:** 2026-10-27

**Authors:** Hélida C. Aquino‐Santos, Jeanne S. Tavares‐Vasconcelos, Anamei Silva‐Reis, Maysa A. R. Brandão‐Rangel, Rayssa T. Morais‐Felix, Fernando A. Santa‐Rosa, André L. L. Bachi, Viviane Soares, Omid Salehian, Claudio R. Frison, Rodolfo P. Vieira, Regiane Albertini

**Affiliations:** ^1^ Post‐graduation Program in Sciences of Human Movement and Rehabilitation Federal University of São Paulo (UNIFESP) São Paulo Brazil; ^2^ School of Physical Education of Military Police of State of São Paulo São Paulo Brazil; ^3^ Laboratory of Pulmonary and Exercise Immunology (LABPEI) Evangelical University of Goias (UniEvangelica) Anápolis Brazil; ^4^ Post‐graduation Program in Health Sciences Santo Amaro University (UNISA) São Paulo Brazil; ^5^ Department of Otorhinolaryngology – Head and Neck Surgery Federal University of Sao Paulo (UNIFESP) São Paulo Brazil; ^6^ Department of Sport Nutrition and Fitness Applied and Science University Tehran Iran; ^7^ Brazilian Institute of Teaching and Research in Pulmonary and Exercise Immunology (IBEPIPE) São Paulo Brazil

**Keywords:** circadian rhythm, cytokine, heart rate variability, inflammation, shiftwork, stress

## Abstract

Chronic shiftwork can impair autonomic cardiovascular regulation and immune homeostasis. However, the interaction between occupational status and physical activity level in military police officers remains incompletely understood. This cross‐sectional factorial study included 102 men stratified according to occupational status and physical activity level: physically active military police officers (PMP; *n* = 26), physically inactive military police officers (PMNP; *n* = 26), physically active civilians (CVP; *n* = 25) and physically inactive civilians (CVNP; *n* = 25). Quality of life, perceived stress, sleepiness, haematological parameters, cytokines and heart rate variability were assessed. Physical activity improved several Short‐form questionnaire (SF‐36) domains, particularly Physical Functioning and General Health. Military police officers demonstrated lower mean normal‐to‐normal intervals and lower high‐frequency power than civilians, indicating differences in resting autonomic cardiovascular modulation associated with occupational status. Serum interleukin‐10 concentrations were significantly lower in military police officers, whereas interleukin‐2, interleukin‐17 and interferon‐γ did not differ significantly according to occupational status. Haemoglobin levels and platelet counts demonstrated significant occupational status × physical activity interactions. Perceived stress scores were lower in military police officers than in civilians. Chronic occupational exposure to shiftwork was associated with altered autonomic cardiovascular regulation and immune regulation in military police officers. Regular physical activity improved quality of life somewhat; however, several physiological alterations remained predominantly associated with occupational status, reinforcing the importance of occupational strategies targeting circadian health and physiological resilience. These findings highlight the physiological complexity of chronic shiftwork exposure in tactical occupational populations.

## Abstract (highlights)

- **What is the central question of this study?**
  Is chronic shiftwork exposure associated with autonomic cardiovascular regulation, immune function and quality of life in military police officers, and is physical activity associated with these outcomes?
- **What is the main finding and its importance?**
  Military police officers exhibited differences in autonomic cardiovascular modulation and lower circulating interleukin‐10 than civilians, whereas interleukin‐2, interleukin‐17 and interferon‐γ did not differ according to occupational status. Physical activity was associated with favourable quality‐of‐life outcomes but did not modify the autonomic or immune findings, suggesting that occupational status is an important correlate of physiological alterations in this population.

## 1 INTRODUCTION

The maintenance of circadian rhythms is essential for physiological homeostasis, including cardiovascular, immune and metabolic functions. The circadian system governs endogenous rhythms every 24 h and synchronizes them with environmental cues through markers such as cortisol secretion, blood pressure variability and immune activity (Morris et al., 2015). Chronic disruption of circadian regulation, such as that observed in shiftworkers, has been consistently associated with increased cardiovascular risk, including hypertension, arterial stiffness, ischaemic events and autonomic imbalance (Nakamura et al., 2022; Owens et al., 2022).

Epidemiological studies have demonstrated a higher prevalence of cardiovascular disease among night‐shiftworkers (Owens et al., 2022). A Brazilian study found that individuals working predominantly at night had a 67% higher risk of elevated cardiovascular risk scores compared with daytime workers, even after adjusting for confounding factors (Silva et al., 2012). Mechanistically, nocturnal light exposure, inversion of the sleep–wake cycle and overactivation of the hypothalamic–pituitary–adrenal axis are believed to drive this increased risk, leading to hypercortisolaemia, elevated blood pressure and a pro‐inflammatory state (Morris et al., 2012, 2016).

Arterial stiffness, in particular, has emerged as an early marker of vascular dysfunction and a predictor of cardiovascular morbidity (Nakamura et al., 2022). Systematic reviews suggest that shiftworkers present greater arterial stiffness compared with non‐shiftworkers, although the data remain heterogeneous, and further longitudinal research is warranted (Nakamura et al., 2022). In addition, markers such as blood pressure variability, C‐reactive protein and interleukins tend to be elevated in chronic shiftworkers (Morris et al., 2015; Owens et al., 2022)

Within the context of military police forces, occupational factors can further exacerbate these cardiovascular risks. Extended periods of sedentary behaviour in patrol vehicles, the use of heavy protective equipment, high occupational stress and repeated exposure to violent environments all contribute to an unfavourable cardiometabolic profile (Braga Filho et al., 2014; França et al., 2011). Local studies indicate that >80% of Brazilian military police officers spend >180 min/day in a seated position, with a high prevalence of dyslipidaemia and hypertension (Braga Filho et al., 2014; França et al., 2011). Furthermore, autonomic dysfunction has been reported among overweight officers, with limited evidence of protective effects from routine physical activity (Carvalho et al., 2018).

Nevertheless, regular physical activity plays a well‐established protective role in cardiovascular health by enhancing insulin sensitivity, endothelial function and autonomic balance. In shiftworkers, exercise might mitigate some of the adverse effects associated with such conditions (Nakamura et al., 2022; Scheer et al., 2009). However, data from studies conducted among Brazilian military police indicate insufficient levels of physical activity; in one cohort from Minas Gerais, 58% of officers were classified as sedentary or insufficiently active, with a significant correlation with burnout syndrome (Santos et al., 2023). Among federal highway police officers, physical inactivity was also closely linked to metabolic syndrome and poor quality‐of‐life indicators (Braga Filho et al., 2014).

Despite growing interest in occupational cardiovascular health, crucial gaps remain in the literature, particularly regarding the combined effects of chronic shiftwork and circadian rhythm disruption and physical activity levels in law enforcement personnel. In our previous study, we demonstrated that chronic shiftwork was associated with impaired pulmonary function and dysregulated immune responses in shiftworkers (Aquino‐Santos et al., 2020). Building upon these findings, the aim of the present study was to investigate cardiovascular responses in Brazilian military police officers exposed to circadian rhythm alterations, with a particular focus on the potential modulatory role of physical activity.

## 2 MATERIALS AND METHODS

### 2.1 Ethical approval

This study was conducted in accordance with the ethical principles of the *Declaration of Helsinki* (2013 revision). The study protocol was approved by the Institutional Research Ethics Committee from Brazil University (registration number 3.020.830), and all participants provided verbal and written informed consent prior to participation.

### 2.2 Participants and study design

A total of 102 male volunteers were recruited, including 52 military police officers [26 active (PMP group) and 26 sedentary (PMNP group)] from the Military Police Battalion of São José dos Campos, São Paulo, Brazil, and 50 non‐military individuals [25 active (CVP group) and 25 sedentary (CVNP group)], referred to as civilians in the present study. In both groups, participants were stratified according to physical activity status, measured by the International Physical Activity Questionnaire (IPAQ) (Graham et al., 2019). Tables 1 and 2 show the clinical characteristics of policemen (active and sedentary) and civilians (active and sedentary), respectively. Additional pairwise comparisons of clinical and physiological characteristics between physically inactive military police officers and physically inactive civilians, and between physically active military police officers and physically active civilians, are provided in Tables 4 and 5, respectively. All military police officers followed the same duty–recovery cycle, consisting of 24 h consecutive on duty followed by 48 h consecutively off duty. To standardize the timing of assessments relative to occupational exposure, all police officers were evaluated only after completion of the full 48 h off‐duty period following their preceding 24 h duty period. Assessments were performed between 08:00 and 11:00 h. Thus, all military police participants were evaluated at the same point in the duty–recovery cycle and within the same morning assessment window.

**TABLE 1:** Clinical and physiological characteristics of physically active and inactive police officers.

Variable	Physically active police officers	Physically inactive police officers	*P*‐value	95% Confidence interval
Age, years	35.92 ± 6.22	38.73 ± 6.79	0.1412	−6.584 to 0.9684
Height, m	177.89 ± 6.56	177.59 ± 8.14	0.8870	−3.907 to 4.504
Body mass, kg	85.31 ± 11.81	91.08 ± 22.54	0.2624	−16.00 to 4.463
Body mass index, kg/m^2^	26.86 ± 3.52	28.58 ± 5.28	0.1919	−4.325 to 0.8930
Waist circumference, cm	94.99 ± 7.89	101.99 ± 15.49	0.0542	−14.15 to 0.1400
Body fat, %	24.87 ± 5.72	30.10 ± 7.21	0.0065	−8.935 to −1.528
Lean mass, %	75.12 ± 5.72	69.81 ± 7.13	0.4588	−52.84 to 24.22
Total body water, %	55.21 ± 4.27	50.57 ± 5.30	0.0020	1.782 to 7.498
Fat‐free mass hydration, %	73.48 ± 2.01	72.43 ± 0.91	0.0223	0.1545 to 1.936
Extracellular water, %	42.24 ± 1.13	42.25 ± 1.15	0.7441	−0.5598 to 0.7782
Intracellular water, %	57.69 ± 1.15	57.91 ± 1.16	0.5055	−0.8825 to 0.4410
Phase angle, 50 Hz	10.24 ± 0.62	9.49 ± 0.80	0.0006	0.3393 to 1.161
Systolic blood pressure, mmHg	123.00 ± 10.51	134.76 ± 21.42	0.0194	−21.56 to −1.975
Diastolic blood pressure, mmHg	71.37 ± 8.64	81.23 ± 13.04	0.0036	−16.34 to −3.370
Pulse pressure, mmHg	50.48 ± 9.76	53.53 ± 12.75	0.3506	−9.586 to 3.469
Resting heart rate, beats/min	66.32 ± 10.87	68.11 ± 10.07	0.5498	−7.792 to 4.201
Maximal inspiratory pressure, cmH_2_O	−122.32 ± 22.21	−132.30 ± 19.12	0.1342	−2.897 to 20.97
Maximal expiratory pressure, cmH_2_O	123.40 ± 21.67	127.50 ± 23.13	0.6296	−15.82 to 9.670
Peak expiratory flow, L/min	623.20 ± 95.26	615.00 ± 104.74	0.6175	−42.70 to 71.16
Fractional exhaled nitric oxide, ppb	13.36 ± 8.66	21.69 ± 18.04	0.0453	−16.49 to −0.1721
Handgrip strength, right, kg	51.28 ± 14.13	46.24 ± 9.22	0.2840	−2.218 to 7.392
Handgrip strength, left, kg	50.00 ± 15.16	43.61 ± 9.07	0.1214	−1.016 to 8.369

**TABLE 2:** Clinical and physiological characteristics of physically active and inactive civilians.

Variable	Physically active civilians	Physically inactive civilians	*P*‐value	95% Confidence interval
Age, years	35.92 ± 6.22	38.73 ± 6.79	0.6862	−5.952 to 3.952
Height, m	177.89 ± 6.56	177.59 ± 8.14	0.0614	−0.1497 to 6.054
Body mass, kg	85.31 ± 11.81	91.08 ± 22.54	0.1978	−2.441 to 11.47
Body mass index, kg/m^2^	26.86 ± 3.52	28.58 ± 5.28	0.4069	−1.178 to 2.853
Waist circumference, cm	94.99 ± 7.89	101.99 ± 15.49	0.8077	−6.527 to 5.112
Body fat, %	24.87 ± 5.72	30.10 ± 7.21	0.9836	−3.369 to 3.439
Lean mass, %	75.12 ± 5.72	69.81 ± 7.13	0.9968	−3.402 to 3.415
Total body water, %	55.21 ± 4.27	50.57 ± 5.30	0.7876	−2.300 to 3.016
Fat‐free mass hydration, %	73.48 ± 2.01	72.43 ± 0.91	0.2024	−0.2989 to 1.371
Extracellular water, %	42.24 ± 1.13	42.25 ± 1.15	0.2425	−0.2412 to 0.9293
Intracellular water, %	57.69 ± 1.15	57.91 ± 1.16	0.2259	−0.9439 to 0.2291
Phase angle, 50 Hz	10.24 ± 0.62	9.49 ± 0.80	0.1839	−0.3199 to 1.618
Systolic blood pressure, mmHg	123.00 ± 10.51	134.76 ± 21.42	0.0189	1.235 to 13.10
Diastolic blood pressure, mmHg	71.37 ± 8.64	81.23 ± 13.04	0.2201	−1.967 to 8.308
Pulse pressure, mmHg	50.48 ± 9.76	53.53 ± 12.75	0.5661	−4.172 to 7.532
Resting heart rate, beats/min	66.32 ± 10.87	68.11 ± 10.07	0.3641	−10.10 to 3.781
Maximal inspiratory pressure, cmH_2_O	−122.32 ± 22.21	−132.30 ± 19.12	0.5727	−19.99 to 11.19
Maximal expiratory pressure, cmH_2_O	123.40 ± 21.67	127.50 ± 23.13	0.5385	−10.34 to 19.54
Peak expiratory flow, L/min	623.20 ± 95.26	615.00 ± 104.74	0.5563	−38.35 to 70.35
Fractional exhaled nitric oxide, ppb	25.08 ± 16.19	18.92 ± 11.52	0.1354	−2.004 to 14.32
Handgrip strength, right, kg	51.28 ± 14.13	46.24 ± 9.22	0.1434	−1.208 to 8.048
Handgrip strength, left, kg	50.00 ± 15.16	43.61 ± 9.07	0.0753	−0.3755 to 7.385

### 2.3 Sample characterization

Physically active military police officers performed structured physical activity as part of their occupational routine, with an average frequency of three sessions per week and a duration of ∼60 min per session. Training sessions were supervised by the platoon sergeant and included cardiorespiratory exercises, such as running on the battalion's running field, and/or resistance training conducted in the battalion's gym facilities. Physically active civilian participants reported engaging in regular structured physical activity, as determined through structured interviews, with a minimum duration of 60 min per session and a minimum frequency of three sessions per week. These activities were performed in settings such as gyms, parks or other outdoor environments and included cardiorespiratory exercises (e.g., running) and/or resistance training in private fitness centres. For both military police officers and civilians, physical activity levels were additionally assessed using the IPAQ. The duration of professional activity was also recorded. Physically active military police officers had 6.84 ± 1.43 years of professional service (median, 7 years; range, 5–10 years), whereas physically inactive military police officers had 6.96 ± 1.61 years of professional service (median, 7 years; range, 5–11 years). The corresponding duration of professional activity was 7.28 ± 1.78 years (median, 7 years; range, 5–11 years) in physically active civilians and 6.96 ± 1.54 years (median, 7 years; range, 5–10 years) in physically inactive civilians.

### 2.4 Sample size calculation

A sample size justification was calculated for a 2 × 2 factorial design, considering two independent fixed factors: occupational status (civilian vs. military police) and physical activity status (physically active vs. physically inactive). The calculation was based on the detection of a moderate interaction effect between occupational status and physical activity status, because this interaction was central to the study hypothesis. Based on an expected partial eta‐squared (η_p_^2^) of 0.076, corresponding to Cohen's *f* = 0.287, with α = 0.05, statistical power of 80%, numerator degrees of freedom = 1, and four independent groups, the minimum required sample size was estimated at 98 participants, corresponding to ∼25 individuals per group. Considering possible data loss, 102 male volunteers were recruited and allocated into four groups: physically active military police officers (*n* = 26), physically inactive military police officers (*n* = 26), physically active civilians (*n* = 25) and physically inactive civilians (*n* = 25). Therefore, the final sample size exceeded the minimum required number and was considered adequate to detect moderate occupational status × physical activity interactions in the evaluated outcomes.

### 2.5 Timing and standardization of assessments

All participants were evaluated during a standardized morning period between 08:00 and 11:00 h. The same assessment time window was applied to military police officers and civilian participants. Military police officers were evaluated during an off‐duty/rest period and were not assessed during an occupational shift. All questionnaires and physiological assessments, including cardiovascular measurements, heart rate variability (HRV), fractional exhaled nitric oxide (FeNO), and blood collection for haematological and immunological analyses and 11‐desoxycortisol determination, were performed during the same morning assessment session. This standardized protocol was adopted to minimize variability associated with clock time and acute occupational shift exposure.

### 2.6 Clinical characterization tests

Only men were recruited for this study. Age, weight, height, body mass index, abdominal circumference, body composition by bioelectrical impedance using multifrequency octopolar bioimpedance equipment, Bioscan 920 II S^®^ (Maltron Int^®^, UK), including fat mass (as a percentage), lean mass (as a percentage), total body water (as a percentage), water in lean mass (as a percentage), extracellular water (as a percentage), intracellular water (as a percentage) and phase angle (in degrees), were measured (Aquino‐Santos et al., 2020). In addition, the health status was recorded by existence of chronic diseases, smoking and medications in use. These results are displayed in Table 3. Resting blood pressure was measured using a validated automated oscillometric digital monitor (HEM‐7122^®^, Omron Healthcare Co.^®^, Ltd, Kyoto, Japan), following standardized measurement procedures (Rachid‐Wolpp et al., 2025). Pulse pressure (PP) was calculated as the difference between systolic and diastolic blood pressure (Rachid‐Wolpp et al., 2025). Maximal inspiratory pressure (MIP) and maximal expiratory pressure (MEP) were measured using an analog manovacuometer (Murenas^®^, Minas Gerais, Brazil), with values expressed in centimetres of water, according to European Respiratory Society standardizations (Aquino‐Santos et al., 2020; Moura‐Maia et al., 2026; Rachid‐Wolpp et al., 2025). Handgrip strength was assessed from both hands using a digital dynamometer (TMax DM‐100, LE Medical^®^, Sao Paulo, Brazil). The results were expressed in kilograms of force (Moura‐Maia et al., 2026). Peak expiratory flow (PEF) was measured using a portable peak flow meter (Medicate^®^, Sao Paulo, Brazil), with values expressed in litres per minute, according to European Respiratory Society/American Thoracic Society recommendations (Graham et al., 2019).

**TABLE 3:** Smoking status, diseases, and medication use.

Variable	Active civilians	Inactive civilians	Active police officers	Inactive police officers
Smokers, *n*	2	0	2	3
Ex‐smokers, years	3	2	2	3
Diabetes, *n*	0	3	0	3
Dyslipidaemia, *n*	0	2	1	1
Hypertension, *n*	0	3	2	4
Bronchitis, *n*	1	0	1	3
Asthma, *n*	3	0	0	1
Rhinosinusitis, *n*	2	5	1	0
Atopic dermatitis, *n*	0	0	1	0
Gastroesophageal reflux disease, *n*	0	0	2	2
Antihypertensive drugs, *n*	1	2	3	5
Proton pump inhibitors, *n*	0	0	2	1
Statins, *n*	1	3	0	0
β_2_‐Agonists, *n*	1	0	0	0
Inhaled corticosteroids, *n*	1	0	0	0
Selective serotonin reuptake inhibitors, *n*	1	1	0	0
Biguanides, *n*	0	2	0	0
Thyroid hormones, *n*	0	1	0	0

**TABLE 4:** Clinical and physiological characteristics of inactive police officers versus inactive civilians.

Variable	Physically inactive police officers	Physically inactive civilians	*P*‐value	95% Confidence interval
Age, years	38.73 ± 6.79	34.00 ± 9.42	0.0529	−0.06729 to 9.529
Height, m	177.59 ± 8.14	174.18 ± 6.00	0.1020	−0.7082 to 7.543
Body mass, kg	91.08 ± 22.54	80.29 ± 13.69	0.0493	0.02372 to 21.56
Body mass index, kg/m^2^	28.58 ± 5.28	26.16 ± 3.86	0.0803	−0.3064 to 5.149
Waist circumference, cm	101.99 ± 15.49	93.28 ± 10.72	0.0304	0.8507 to 16.57
Body fat, %	30.10 ± 7.21	31.45 ± 0.09	0.0243	0.6191 to 8.596
Lean mass, %	69.81 ± 7.13	68.55 ± 0.09	0.4570	−25.22 to 55.16
Total body water, %	50.57 ± 5.30	49.24 ± 0.26	0.0164	−6.746 to −0.7126
Fat‐free mass hydration, %	72.43 ± 0.91	71.84 ± 0.48	0.2286	−1.144 to 0.2806
Extracellular water, %	42.25 ± 1.15	41.03 ± 0.46	0.1373	−0.1705 to 1.198
Intracellular water, %	57.91 ± 1.16	58.95 ± 0.46	0.2279	−1.091 to 0.2669
Phase angle, 50 Hz	9.49 ± 0.80	10.86 ± 0.84	0.5801	−1.043 to 0.5907
Systolic blood pressure, mmHg	134.76 ± 21.42	120.00 ± 17.00	0.0073	4.045 to 24.62
Diastolic blood pressure, mmHg	81.23 ± 13.04	76.00 ± 8.00	0.0083	2.522 to 16.20
Pulse pressure, mmHg	53.53 ± 12.75	44.00 ± 9.00	0.4516	−4.389 to 9.706
Resting heart rate, beats/min	68.11 ± 10.07	72.95 ± 8.19	0.0421	−10.77 to −0.1998
Maximal inspiratory pressure, cmH_2_O	−132.30 ± 19.12	−90.00 ± 10.00	0.1751	−23.41 to 4.391
Maximal expiratory pressure, cmH_2_O	127.50 ± 23.13	85.00 ± 15.00	0.1112	−2.810 to 26.21
Peak expiratory flow, L/min	615.00 ± 104.74	630.00 ± 105.84	0.8067	−67.92 to 53.12
Fractional exhaled nitric oxide, ppb	21.69 ± 18.04	18.92 ± 11.52	0.5263	−5.965 to 11.51
Handgrip strength, right, kg	46.24 ± 9.22	52.00 ± 12.00	0.6527	−4.292 to 6.784
Handgrip strength, left, kg	43.61 ± 9.07	49.00 ± 7.00	0.4667	−3.033 to 6.514

**TABLE 5:** Clinical and physiological characteristicsof active police officers versus active civilians.

Variable	Physically active police officers	Physically active civilians	*P*‐value	95% Confidence interval
Age, years	35.92 ± 6.22	32.91 ± 7.33	0.1401	−0.9971 to 6.843
Height, m	177.89 ± 6.56	177.13 ± 4.57	0.6396	−2.498 to 4.026
Body mass, kg	85.31 ± 11.81	84.80 ± 9.94	0.8707	−5.775 to 6.798
Body mass index, kg/m^2^	26.86 ± 3.52	27.00 ± 2.94	0.8887	−2.021 to 1.757
Waist circumference, cm	94.99 ± 7.89	92.58 ± 9.04	0.3299	−2.520 to 7.344
Body fat, %	24.87 ± 5.72	25.53 ± 5.08	0.6725	−3.773 to 2.456
Lean mass, %	75.12 ± 5.72	74.46 ± 5.08	0.6725	−2.456 to 3.773
Total body water, %	55.21 ± 4.27	54.66 ± 3.94	0.6593	−1.955 to 3.060
Fat‐free mass hydration, %	73.48 ± 2.01	73.40 ± 1.35	0.8757	−0.9131 to 1.068
Extracellular water, %	42.24 ± 1.13	42.22 ± 0.80	0.3349	−0.2975 to 0.8557
Intracellular water, %	57.69 ± 1.15	57.96 ± 0.80	0.3406	−0.8507 to 0.3001
Phase angle, 50 Hz	10.24 ± 0.62	10.37 ± 1.43	0.6924	−0.7575 to 0.5073
Systolic blood pressure, mmHg	123.00 ± 10.51	127.60 ± 8.32	0.1033	−10.18 to 0.9751
Diastolic blood pressure, mmHg	71.37 ± 8.64	75.04 ± 7.45	0.1261	−8.403 to 1.073
Pulse pressure, mmHg	50.48 ± 9.76	52.56 ± 8.08	0.4236	−7.266 to 3.106
Resting heart rate, beats/min	66.32 ± 10.87	70.34 ± 15.40	0.2756	−11.64 to 3.400
Maximal inspiratory pressure, cmH_2_O	−122.32 ± 22.21	−127.20 ± 19.12	0.5674	−9.801 to 17.66
Maximal expiratory pressure, cmH_2_O	123.40 ± 21.67	120.40 ± 24.07	0.5413	−9.133 to 17.18
Peak expiratory flow, L/min	623.20 ± 95.26	638.40 ± 79.38	0.7165	−59.66 to 41.33
Fractional exhaled nitric oxide, ppb	13.36 ± 8.66	25.08 ± 16.19	0.0029	−19.24 to −4.203
Handgrip strength, right, kg	51.28 ± 14.13	48.50 ± 5.33	0.8219	−3.264 to 4.091
Handgrip strength, left, kg	50.00 ± 15.16	45.68 ± 6.02	0.3132	−1.864 to 5.688

### 2.7 Inclusion and exclusion criteria

Inclusion criteria were male individuals aged between 25 and 59 years, classified as either physically active or inactive; for the military group, with a minimum of 5 years of professional service; and for the civilian group, a regular work schedule of ∼44 h/week, during daytime (Monday–Friday), for ≥5 years. Exclusion criteria included the presence of neuromuscular or infectious diseases that could impair participation in the proposed assessments; a prior history of pulmonary and/or cardiovascular diseases; and irregular engagement and <1 year of regular practice of physical activity, for the active volunteers.

### 2.8 Evaluation of perceived stress

Perceived stress was assessed using the Perceived Stress Scale questionnaire (Aquino‐Santos et al., 2020).

### 2.9 Evaluation of sleepiness

Daytime sleepiness was assessed using the Epworth Sleepiness Scale (Aquino‐Santos et al., 2020; Bertolazi et al., 2009).

### 2.10 Evaluation of physical activity/inactivity levels

Physical activity levels were assessed using the short version of the IPAQ (Aquino‐Santos et al., 2020; Lee et al., 2011).

### 2.11 Fractional exhaled nitric oxide

FeNO was measured using the NObreath^®^ FeNO monitor (Bedfont Scientific Ltd, UK), which uses electrochemical sensor technology for the detection of nitric oxide in exhaled breath. FeNO values were expressed in parts per billion (Aquino‐Santos et al., 2020; Moura‐Maia et al., 2026).

### 2.12 Systemic inflammation and immune response

Five millilitres of venous blood was collected into vacuum tubes containing EDTA‐K_2_ as anticoagulant. Blood samples were collected between 08:00 and 11:00 h during the standardized assessment session. For all military police officers, sampling occurred after completion of the full 48 h off‐duty period following the preceding 24 h duty period, whereas civilians were assessed within the same morning time window. Thus, blood collection was standardized according to clock time and, for military police officers, to the same point in the duty–recovery cycle. Haematological parameters, including leucocyte, erythrocyte and platelet counts, were analysed using an automated haematology analyser, Sysmex XS‐800i^®^ (Sysmex^®^, Germany) (Aquino‐Santos et al., 2020; Moura‐Maia et al., 2026). Following haematological analysis, blood samples were centrifuged at 1000*g* for 7 min at 4°C, and the obtained serum was stored until further analysis. Serum concentrations of interleukin (IL)‐2, IL‐10, IL‐17 and interferon‐γ (IFN‐γ) were quantified using DuoSet^®^ ELISA kits (R&D Systems^®^), with absorbance readings obtained using the SpectraMax i3^®^ microplate reader (Molecular Devices^®^, CA, USA) (Aquino‐Santos et al., 2020; Moura‐Maia et al., 2026). Additionally, serum 11‐desoxycortisol concentrations were determined by chemiluminescence (Aquino‐Santos et al., 2020).

### 2.13 Assessment of HRV

HRV was assessed through resting ECG recordings using a digital 12‐lead ECG (ECGPC^®^, Electronic Brazilian Technology‐TEB^®^, Brazil). The system enables non‐invasive acquisition of high‐resolution cardiac electrical signals via adhesive surface electrodes positioned according to standard lead placement guidelines. All assessments were conducted in a quiet, temperature‐controlled environment, with participants instructed to avoid caffeine, alcohol and vigorous physical activity for ≥24 h prior to testing. Recordings were performed with participants in the supine position, breathing spontaneously, and instructed to remain awake, calm and motionless throughout the procedure to minimize external influences on autonomic modulation. Initially, a 1 min ECG recording was obtained to allow signal stabilization, followed by a continuous 10 min recording for HRV analysis. Data acquisition was performed in standardized resting conditions, ensuring consistency across all participants. ECG signals were processed using the manufacturer's dedicated software (ECGPC^®^, Electronic Brazilian Technology‐TEB^®^, Brazil), which automatically detected R‐wave peaks and computed beat‐to‐beat intervals. All recordings were inspected visually, and artefacts and ectopic beats were identified and excluded when necessary. Only normal‐to‐normal (NN) intervals were considered for HRV analysis. HRV indices were calculated in accordance with the recommendations of the Task Force of the European Society of Cardiology and the North American Society of Pacing & Electrophysiology (1996). Time‐domain parameters included the mean NN interval (in milliseconds), root mean square of successive differences (RMSSD, in milliseconds) and the percentage of adjacent NN intervals differing by >50 ms (pNN50, as a percentage). Frequency‐domain analysis included low‐frequency power (LF, 0.04–0.15 Hz) and high‐frequency power (HF, 0.15–0.40 Hz), expressed in normalized units (n.u.). HF power was considered predominantly related to parasympathetic modulation, whereas LF power was interpreted cautiously because it reflects contributions from both sympathetic and parasympathetic mechanisms. The LF/HF ratio was not considered a primary outcome because its interpretation as a direct measure of sympathovagal balance is physiologically controversial. Importantly, the 10 min recordings were designed to characterize short‐term resting cardiac autonomic modulation and not circadian rhythmicity of HRV.

### 2.14 Statistical analysis

Statistical analyses were performed using IBM SPSS Statistics^®^ (v.27.0; IBM Corp.^®^, Armonk, NY, USA), and graphical representations were generated using GraphPad Prism^®^ 8.0 (GraphPad Software^®^, San Diego, CA, USA). Initially, data distribution was assessed using the Shapiro–Wilk test, and homogeneity of variances was evaluated using Levene's test. Continuous variables are presented as the mean ± SD for descriptive purposes, together with the median and interquartile range (IQR) when appropriate. The study followed a 2 × 2 cross‐sectional factorial design including two independent factors: occupational status (civilian vs. military police) and physical activity status (physically active vs. physically inactive). Therefore, the primary inferential statistical approach consisted of a two‐way ANOVA, with occupational status and physical activity status entered as fixed between‐subject factors. The interaction term (occupational status × physical activity status) was included in the model to determine whether the association between physical activity and the evaluated outcomes differed according to occupational category. Main effects of occupational status and physical activity status, in addition to the occupational status × physical activity interaction, were evaluated within this factorial model. When significant main or interaction effects were identified, Bonferroni‐adjusted *post hoc* pairwise comparisons were performed to control for multiple testing. In addition, exploratory pairwise comparisons between groups were conducted using Student's unpaired *t*‐tests for normally distributed variables with homogeneity of variances. For variables violating parametric assumptions, the Mann–Whitney *U*‐test was additionally performed as a complementary non‐parametric pairwise approach. Importantly, Mann–Whitney *U*‐tests were not used to assess factorial main effects or occupational status × physical activity interactions; these effects were evaluated exclusively within the two‐way ANOVA framework. Effect sizes for ANOVA models were expressed as η_p_^2^ and interpreted as small (≥0.01), moderate (≥0.06) or large (≥0.14). For pairwise comparisons, Cohen's *d* was calculated and interpreted as small (≥0.20), moderate (≥0.50) or large (≥0.80). For non‐parametric analyses, rank‐biserial correlation (*r*) was used as an estimate of effect size. As a sensitivity analysis, cytokine outcomes displaying extreme observations were reanalysed after exclusion of extreme values, defined a priori as observations lying more than three IQRs (3 × IQR) below the first quartile or above the third quartile. The 2 × 2 factorial ANOVA was then repeated using the same occupational status and physical activity factors to assess the robustness of the primary findings. All tests were two tailed, and statistical significance was established at *P* < 0.05.

## 3 RESULTS

### 3.1 Quality of life, sleepiness, perceived stress, physical activity and 11‐desoxycortisol

Physical activity significantly influenced several SF‐36 domains, particularly those related to physical functioning and general health perception (Figure 1). The Short‐form questionnaire (SF‐36) Physical Functioning domain (Figure 1a) demonstrated a significant main effect of physical activity status [*F*(1,98) = 9.04, *P* = 0.003, η_p_^2^ = 0.084], indicating higher scores in physically active participants independently of occupational status. Physically active civilians (CVP) and physically active military police officers (PMP) presented higher Physical Functioning scores than their physically inactive counterparts. Bonferroni‐adjusted *post hoc* analysis demonstrated significantly higher scores in CVP compared with physically inactive military police officers (PMNP; *P* = 0.035; *d* = 0.81). No significant occupational status effect or occupational status × physical activity interaction was observed for this domain.

**FIGURE 1:**
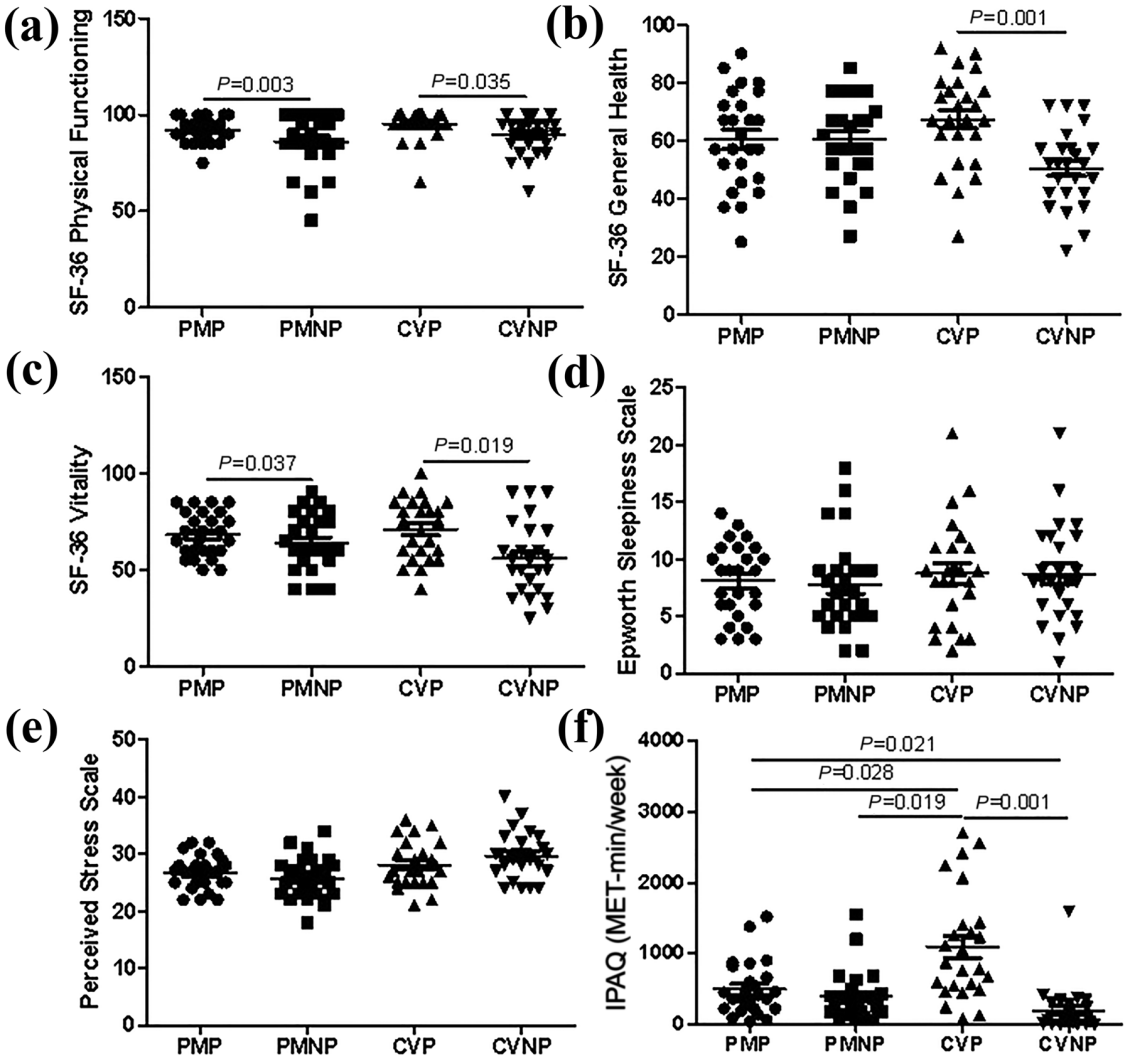
Scatter dot plots showing individual values and group means (horizontal bars ± SD) for physically active military police officers (PMP), physically inactive military police officers (PMNP), physically active civilians (CVP) and physically inactive civilians (CVNP). (a) SF‐36 Physical Functioning domain score. (b) SF‐36 General Health domain score. (c) SF‐36 Vitality domain score. (d) Epworth sleepiness scale score. (e) Perceived stress scale score. (f) International physical activity questionnaire (IPAQ) physical activity levels expressed as MET‐min/week. Statistical analysis was performed using two‐way ANOVA considering occupational status (civilian vs. military police) and physical activity status (physically active vs. physically inactive), followed by Bonferroni‐adjusted *post hoc* comparisons when appropriate. Significant pairwise comparisons are indicated in the figure.

General Health scores (Figure 1b) demonstrated a significant main effect of physical activity status [*F*(1,98) = 7.64, *P* = 0.007, η_p_^2^ = 0.072] and a significant occupational status × physical activity interaction [*F*(1,98) = 8.02, *P* = 0.006, η_p_^2^ = 0.076]. CVP presented markedly higher General Health scores compared with physically inactive civilians (CVNP; *P* = 0.001; *d* = 1.15), whereas no significant differences were observed between physically active and inactive military police officers. These findings indicate that the beneficial effects of physical activity on perceived general health were more evident among civilians than among military police officers.

Likewise, Vitality scores (Figure 1c) were significantly influenced by the occupational status × physical activity interaction [*F*(1,98) = 10.32, *P* = 0.002, η_p_^2^ = 0.095]. CVP demonstrated significantly higher Vitality scores compared with CVNP (*P* = 0.019; *d* = 0.88), and PMNP also presented higher values than CVNP (*P* = 0.037; *d* = 0.80). No additional pairwise comparisons remained significant after Bonferroni correction. In contrast, Role Physical, Bodily Pain and Role Emotional domains did not demonstrate significant effects of occupational status or physical activity status or interaction effects (all *P *> 0.05), although moderate effect sizes suggested a tendency towards better scores among physically active civilians.

Social Functioning scores demonstrated a significant main effect of occupational status [*F*(1,98) = 5.48, *P* = 0.021, η_p_^2^ = 0.053], with military police officers presenting overall higher values than civilians. Although the occupational status × physical activity interaction approached statistical significance (*P* = 0.054), Bonferroni‐adjusted comparisons did not reveal significant pairwise differences among groups. Mental Health scores were significantly influenced by occupational status [*F*(1,92) = 5.58, *P* = 0.020, η_p_^2^ = 0.057] and by the occupational status × physical activity interaction [*F*(1,92) = 8.46, *P* = 0.005, η_p_^2^ = 0.084]. Physically inactive military police officers presented significantly higher Mental Health scores than physically inactive civilians (*P* = 0.010; *d* = 0.96), whereas physical activity status alone did not significantly influence this domain.

Daytime sleepiness, assessed using the Epworth sleepiness scale (Figure 1d), did not differ significantly according to occupational status, physical activity status or their interaction (all *P *> 0.05). Epworth sleepiness scale scores remained relatively similar among all experimental groups.

Perceived Stress Scale scores (Figure 1e) demonstrated a significant main effect of occupational status [*F*(1,98) = 5.16, *P* = 0.025, η_p_^2^ = 0.050], indicating lower perceived stress scores in military police officers compared with civilians. No significant main effect of physical activity status or occupational status × physical activity interaction was observed.

As expected, IPAQ‐derived physical activity levels (Figure 1f) demonstrated a robust main effect of physical activity status [*F*(1,98) = 13.81, *P* < 0.001, η_p_^2^ = 0.124] and a significant occupational status × physical activity interaction [*F*(1,98) = 13.09, *P* < 0.001, η_p_^2^ = 0.118]. CVP presented significantly higher MET‐min/week values than PMP (*P* = 0.019), PMNP (*P* = 0.028) and CVNP (*P* < 0.001). In addition, PMP demonstrated higher physical activity levels than CVNP (*P* = 0.021). These findings confirm the adequacy of group stratification according to habitual physical activity level.

Serum 11‐desoxycortisol concentrations (PMP, 1.88 ± 0.27 ng/mL; PMNP, 1.99 ± 0.46 ng/mL; CVP, 1.58 ± 0.62 ng/mL; and CVNP, 1.79 ± 0.57 ng/mL) demonstrated a significant main effect of occupational status [*F*(1,92) = 4.891, *P* = 0.029, η_p_^2^ = 0.050], with military police officers presenting overall higher values than civilians. No significant effects of physical activity status or interaction effects were observed. Although Bonferroni‐adjusted pairwise comparisons did not identify statistically significant differences among individual groups, PMNP demonstrated numerically higher concentrations compared with CVP.

### 3.2 HRV and autonomic modulation

HRV analysis revealed important differences according to occupational status (Figure 2). RMSSD and pNN50, both predominantly associated with vagal cardiac modulation, did not demonstrate statistically significant main effects of occupational status or physical activity status or occupational status × physical activity interactions (all *P *> 0.05). Nevertheless, moderate effect sizes and numerical trends consistently indicated lower vagally mediated HRV indices among military police officers, particularly in PMNP compared with CVNP.

**FIGURE 2:**
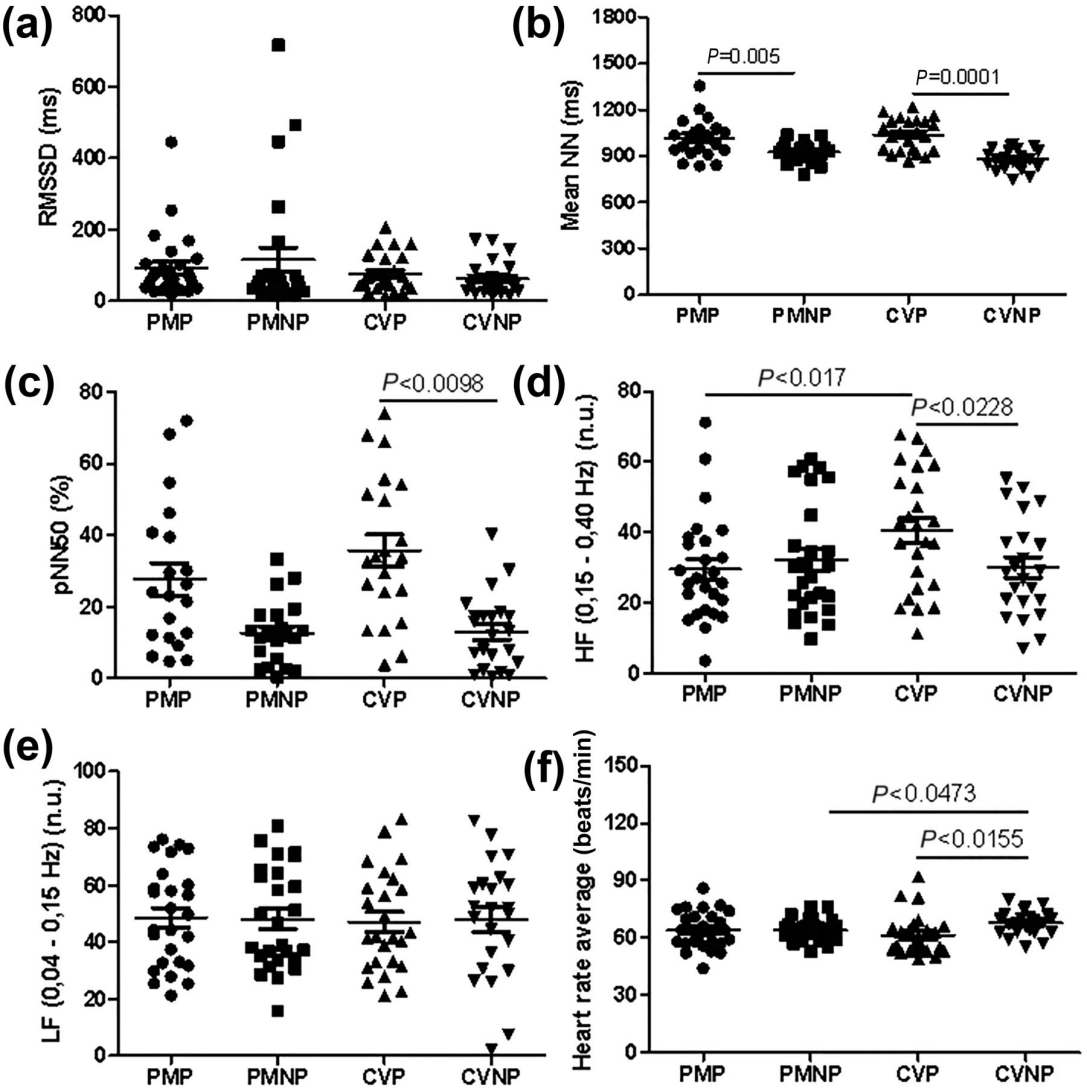
Scatter dot plots showing individual values and group means (horizontal bars ± SD) for physically active military police officers (PMP), physically inactive military police officers (PMNP), physically active civilians (CVP) and physically inactive civilians (CVNP). (a) Root mean square of successive differences (RMSSD; in milliseconds). (b) Mean normal‐to‐normal interval (Mean NN; in milliseconds). (c) Percentage of adjacent NN intervals differing by >50 ms (pNN50; as a percentage). (d) High‐frequency power [HF, 0.15–0.40 Hz; in normalized units (n.u.)]. (e) Low‐frequency power [LF, 0.04–0.15 Hz; in normalized units (n.u.)]. (f) Resting heart rate (in beats per minute). Statistical analysis was performed using two‐way ANOVA considering occupational status and physical activity status. Significant occupational main effects were observed for Mean NN, HF, LF and resting heart rate, whereas RMSSD and pNN50 did not demonstrate significant occupational effects. Bonferroni‐adjusted *post hoc* comparisons were performed when appropriate.

In contrast, Mean NN intervals (Figure 2b) demonstrated a significant main effect of occupational status [*F*(1,96) = 4.88, *P* = 0.029, η_p_^2^ = 0.048], with military police officers presenting lower beat‐to‐beat cardiac intervals than civilians. Likewise, HF power expressed in normalized units (Figure 2d) was significantly lower in military police officers compared with civilians [*F*(1,96) = 5.42, *P* = 0.022, η_p_^2^ = 0.053], whereas LF power (Figure 2e) was significantly lower in military police officers [*F*(1,96) = 5.42, *P* = 0.022, η_p_^2^ = 0.053]. No significant effects of physical activity status or interaction effects were observed for these frequency‐domain parameters.

Resting heart rate demonstrated a significant main effect of occupational status [*F*(1,96) = 6.18, *P* = 0.015, η_p_^2^ = 0.061]. Exploratory pairwise comparisons indicated lower resting heart rate in PMNP than in CVNP, with a large effect size (*d* = 0.89), although this pairwise difference did not remain statistically significant after Bonferroni correction. No significant main effect of physical activity status or occupational status × physical activity interaction was observed. Collectively, these findings indicate that occupational status was associated with differences in resting autonomic cardiovascular modulation in the present cohort. Exploratory pairwise comparisons demonstrated lower resting heart rate values in PMNP compared with CVNP, with a large effect size (*d* = 0.89), although this difference did not remain significant after Bonferroni correction. Collectively, these findings indicate that occupational status exerted a stronger influence on autonomic cardiovascular modulation than physical activity status in the present cohort.

### 3.3 Haematological parameters

Most haematological variables did not differ significantly according to occupational status, physical activity status or occupational status × physical activity interactions. Total leucocyte counts, neutrophils, lymphocytes, monocytes, eosinophils, basophils and red blood cell counts all demonstrated non‐significant factorial effects (all *P *> 0.05), with substantial overlap among groups.

In contrast, haemoglobin (Figure 3a) concentrations demonstrated a significant main effect of occupational status [*F*(1,99) = 6.149, *P* = 0.015, η_p_^2^ = 0.058], indicating overall higher haemoglobin concentrations in civilians than in military police officers. In addition, a significant occupational status × physical activity interaction was identified [*F*(1,99) = 5.033, *P* = 0.027, η_p_^2^ = 0.048]. Bonferroni‐adjusted *post hoc* analysis demonstrated significantly higher haemoglobin concentrations in CVP compared with PMP, whereas no additional pairwise differences reached statistical significance.

**FIGURE 3:**
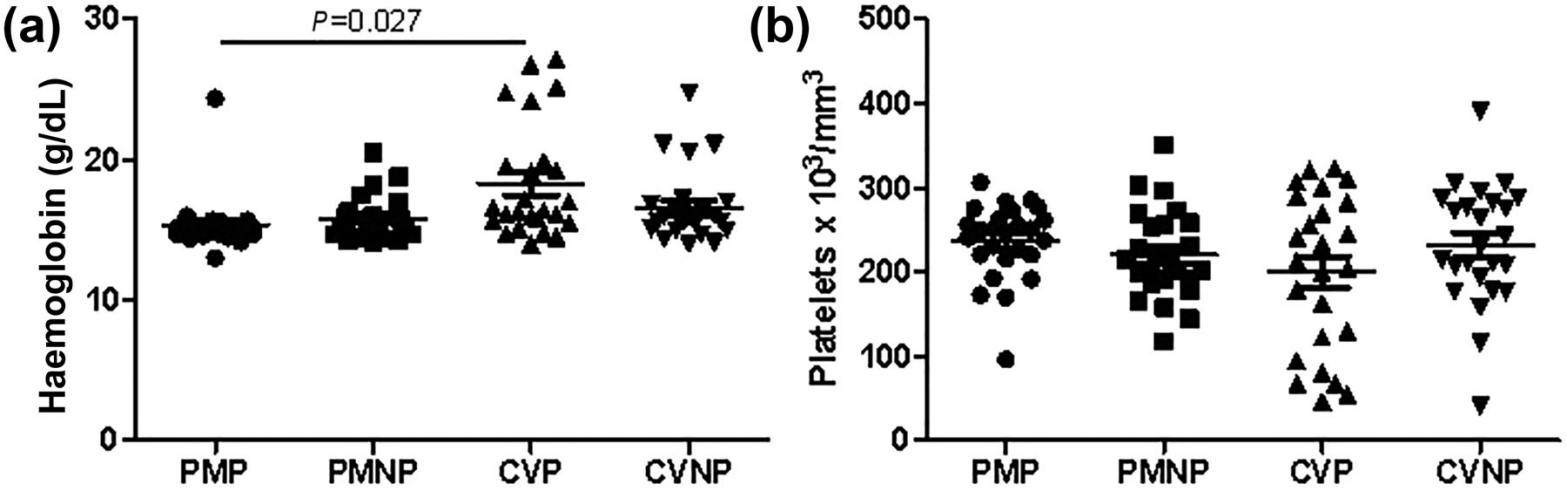
Scatter dot plots showing individual values and group means (horizontal bars ± SD) for physically active military police officers (PMP), physically inactive military police officers (PMNP), physically active civilians (CVP) and physically inactive civilians (CVNP). (a) Haemoglobin concentration (in grams per decilitre). (b) Platelet count (×10^3^ per millimetre cubed). Statistical analysis was performed using two‐way ANOVA considering occupational status (civilian vs. military police) and physical activity status (physically active vs. physically inactive), followed by Bonferroni‐adjusted *post hoc* comparisons when appropriate. Significant pairwise comparisons are indicated in the figure.

Platelet (Figure 3b) counts also demonstrated a significant occupational status × physical activity interaction [*F*(1,99) = 4.053, *P* = 0.047, η_p_^2^ = 0.039], despite the absence of significant main effects of occupational status or physical activity status. However, Bonferroni‐adjusted pairwise comparisons did not identify statistically significant differences among the individual groups.

### 3.4 Cytokines and immune response

Circulating cytokine concentrations demonstrated distinct patterns according to occupational status (Figure 4). Serum IL‐2 concentrations did not demonstrate significant main effects of occupational status [*F*(1,95) = 0.181, *P* = 0.672, η_p_^2^ = 0.002] or physical activity status [*F*(1,95) = 0.140, *P* = 0.709, η_p_^2^ = 0.001], nor an occupational status × physical activity interaction [*F*(1,95) = 0.604, *P* = 0.439, η_p_^2^ = 0.006].

**FIGURE 4:**
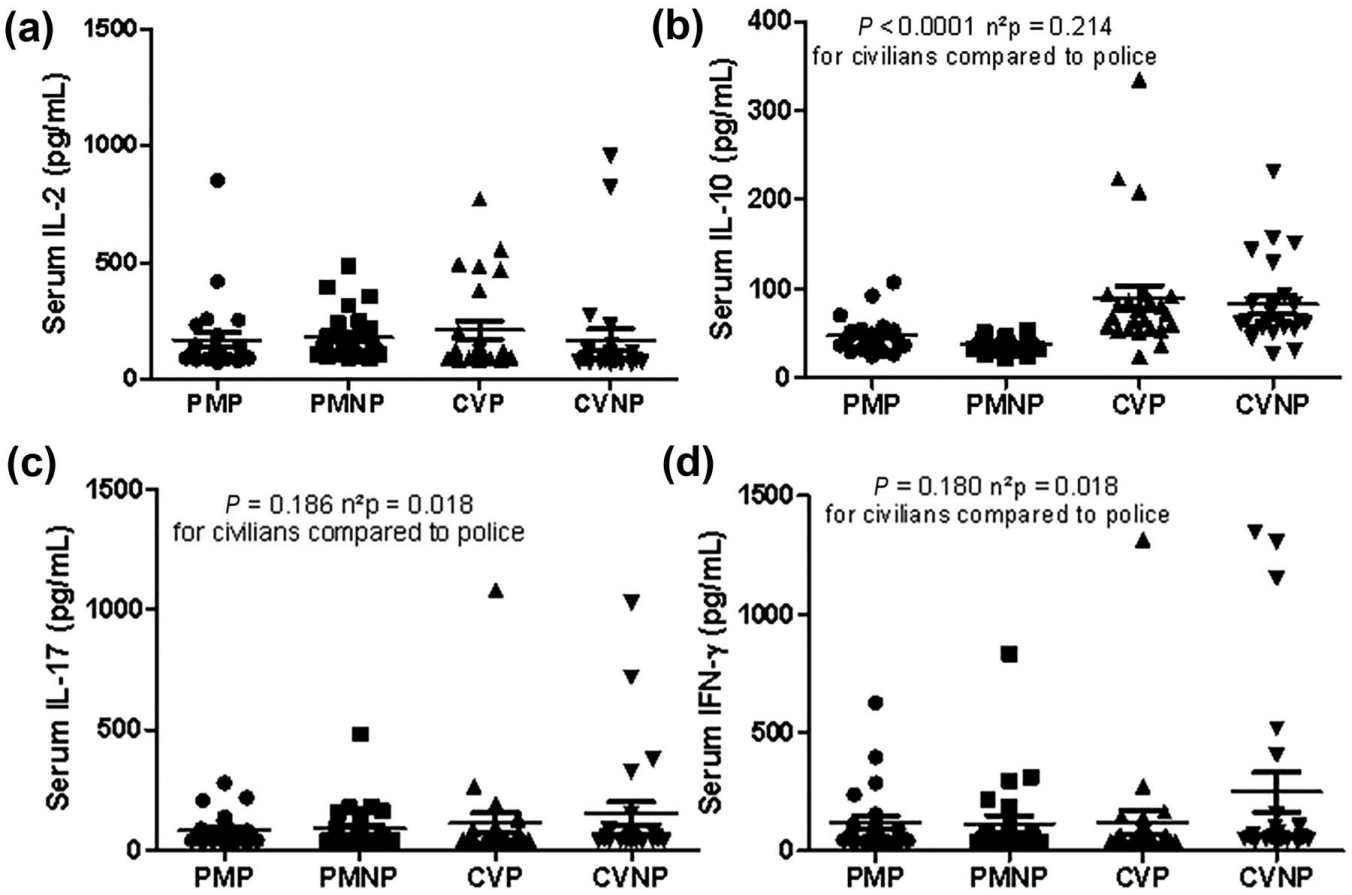
Scatter dot plots showing individual values and group means (horizontal bars ± SD) for physically active military police officers (PMP), physically inactive military police officers (PMNP), physically active civilians (CVP) and physically inactive civilians (CVNP). (a) Serum interleukin‐2 (IL‐2) concentration. (b) Serum interleukin‐10 (IL‐10) concentration. (c) Serum interleukin‐17 (IL‐17) concentration. (d) Serum interferon‐γ (IFN‐γ) concentration. Cytokine concentrations are expressed as picograms per millilitre. Statistical analysis was performed using two‐way ANOVA considering occupational status (civilian vs. military police) and physical activity status (physically active vs. physically inactive). The main effect of occupational status was significant only for IL‐10 (*P* < 0.0001), whereas occupational effects were not significant for IL‐2 (*P* = 0.672), IL‐17 (*P* = 0.186) and IFN‐γ (*P* = 0.180). Bonferroni‐adjusted *post hoc* comparisons were performed when appropriate.

In contrast, serum IL‐10 demonstrated a strong main effect of occupational status [*F*(1,95) = 25.880, *P* < 0.0001, η_p_^2^ = 0.214], with civilians presenting higher concentrations than military police officers. No significant main effect of physical activity [*F*(1,95) = 1.090, *P* = 0.299, η_p_^2^ = 0.011] or occupational status × physical activity interaction [*F*(1,95) = 0.031, *P* = 0.861, η_p_^2^ < 0.001] was observed.

No significant factorial effects were identified for IL‐17. The main effect of occupational status was not significant [*F*(1,95) = 1.773, *P* = 0.186, η_p_^2^ = 0.018], nor were the effects of physical activity [*F*(1,95) = 0.369, *P* = 0.545, η_p_^2^ = 0.004] or occupational status × physical activity interaction [*F*(1,95) = 0.218, *P* = 0.642, η_p_^2^ = 0.002]. Likewise, IFN‐γ demonstrated no significant main effect of occupational status [*F*(1,99) = 1.820, *P* = 0.180, η_p_^2^ = 0.018] or physical activity [*F*(1,99) = 1.433, *P* = 0.234, η_p_^2^ = 0.014] or occupational status × physical activity interaction [*F*(1,99) = 1.560, *P* = 0.215, η_p_^2^ = 0.016].

Sensitivity analyses excluding extreme observations (>3 × IQR) confirmed the robustness of the principal findings. The occupational effect on IL‐10 remained highly significant and increased in magnitude [*F*(1,91) = 39.666, *P* < 0.0001, η_p_^2^ = 0.304]. IL‐17 and IFN‐γ remained non‐significant after exclusion of extreme observations [occupation: IL‐17, *F*(1,87) = 2.005, *P* = 0.160, η_p_^2^ = 0.023; IFN‐γ, *F*(1,90) = 0.676, *P* = 0.413, η_p_^2^ = 0.007]. For IL‐2, an occupational effect emerged in the sensitivity analysis [*F*(1,86) = 6.942, *P* = 0.010, η_p_^2^ = 0.075], although this effect was not present in the primary analysis and should therefore be interpreted as exploratory.

## 4 DISCUSSION

The present findings demonstrate that chronic occupational exposure to shiftwork in military police officers is associated with alterations in cardiovascular autonomic modulation, immune regulation and perceived health outcomes. Importantly, regular physical activity appeared somewhat to attenuate some adverse effects associated with chronic shiftwork, particularly in domains related to quality of life and general health perception. However, several physiological alterations remained predominantly associated with occupational status rather than with physical activity level alone. These findings reinforce the complex interaction between chronic shiftwork, occupational stress, autonomic regulation and systemic immune homeostasis in populations chronically exposed to shiftwork‐related stressors. One of the main findings of the present study was that military police officers demonstrated distinct autonomic cardiovascular modulation compared with civilians, characterized by lower Mean NN intervals and lower HF power. In contrast, RMSSD and pNN50 did not show significant occupational effects, indicating that the observed differences should not be interpreted as evidence of a generalized alteration in parasympathetic modulation. Resting heart rate also showed a significant main effect of occupational status, although exploratory pairwise analysis indicated that the lower resting heart rate observed in physically inactive military police officers compared with physically inactive civilians did not remain significant after Bonferroni correction. Previous investigations have demonstrated that chronic occupational stress might be associated with complex patterns of autonomic regulation rather than a uniform shift towards sympathetic predominance, particularly in populations repeatedly exposed to stressful environments and prolonged vigilance states (Jarczok et al., 2019; Thayer et al., 2010). Repeated exposure to high‐alert occupational conditions has also been associated with alterations in cardiovascular autonomic regulation, potentially reflecting physiological adaptations to recurrent stress exposure (Tobaldini et al., 2017), and changes in autonomic cardiovascular responses have also been reported in tactical and military populations exposed to repeated operational stress (Fonkoue et al., 2021). Therefore, rather than indicating greater vagal modulation, as previously interpreted, the present findings suggest a complex pattern of autonomic regulation associated with occupational status. Given the cross‐sectional design of the present study, however, it is not possible to determine whether these differences represent adaptive responses, adverse autonomic alterations or other consequences of chronic occupational and shiftwork exposure. The finding that physically inactive military police officers presented numerically favourable HRV parameters compared with sedentary civilians is particularly interesting. Although these differences did not remain significant after Bonferroni correction, moderate‐to‐large effect sizes were observed for several autonomic indices. One possible explanation is that military police officers, even when classified as physically inactive according to IPAQ criteria, might still experience higher daily occupational physical demands than civilians, including prolonged standing, operational readiness, rapid response situations and intermittent high‐intensity physical efforts (Violanti et al., 2012). Such occupational demands might partly preserve cardiovascular autonomic conditioning despite insufficient structured exercise practice.

Nevertheless, the autonomic findings should be interpreted cautiously. Chronic exposure to demanding occupational conditions might produce complex cardiovascular adaptations that are not characterized adequately as either simple sympathetic predominance or enhanced vagal modulation (Fonkoue et al., 2021; Jarczok et al., 2019; Thayer et al., 2010; Tobaldini et al., 2017). Moreover, the lower perceived stress reported by police officers might reflect resilience or habituation, but could also be influenced by differences in stress perception or reporting (Fonkoue et al., 2021; Jarczok et al., 2019; Thayer et al., 2010; Tobaldini et al., 2017). Given the cross‐sectional design and the single resting HRV assessment, we cannot determine whether the observed autonomic profile represents adaptive resilience, cumulative occupational strain or longer‐term maladaptive autonomic changes. Longitudinal studies incorporating repeated autonomic, neuroendocrine and psychological assessments are required to distinguish these possibilities. However, an alternative possibility is that this pattern reflects longer‐term autonomic adaptation or reduced physiological responsiveness following prolonged stress exposure, sometimes conceptualized as autonomic exhaustion. The concomitantly lower perceived stress reported by police officers might, likewise, reflect resilience or habituation but could also reflect altered stress perception or reporting. Importantly, the present cross‐sectional findings cannot distinguish between adaptive and potentially maladaptive mechanisms. Longitudinal studies incorporating repeated autonomic, neuroendocrine and psychological assessments are therefore required to determine the physiological trajectory and clinical significance of this autonomic profile.

Regular physical activity significantly improved several SF‐36 domains, particularly Physical Functioning and General Health, confirming the protective effects of exercise on perceived health and functionality. These findings are consistent with recent evidence demonstrating that exercise improves physical and psychological resilience in workers exposed to chronic shiftwork (Bull et al., 2020; da Silva et al., 2014). Importantly, the significant interaction observed for General Health and Vitality suggests that the beneficial effects of physical activity were more pronounced among civilians than among military police officers. This finding might indicate that chronic occupational stress associated with police work somwhat blunts the beneficial effects of regular physical activity on subjective well‐being and vitality perception. Previous studies suggested that persistent sympathetic activation, sleep restriction and psychological hypervigilance might impair recovery mechanisms and reduce the psychophysiological benefits typically induced by exercise (Wirth et al., 2017).

Unexpectedly, military police officers presented lower perceived stress scores compared with civilians. At first glance, this result might appear contradictory, considering the stressful nature of police work. However, previous studies have demonstrated that chronic exposure to stressful occupational environments might induce habituation phenomena and alter stress perception, particularly in tactical and emergency‐response professions (Purba and Demou, 2019). In addition, cultural and behavioural characteristics among military personnel might influence stress self‐reporting, potentially leading to underestimation of perceived psychological burden (Karaffa & Koch, 2016). The lower perceived stress scores observed in police officers therefore do not necessarily indicate lower biological stress exposure.

Another relevant finding of the present study involves the immune profile observed in military police officers. Although total leucocyte counts and differential cell counts did not differ significantly among groups, occupational status was significantly associated with circulating IL‐10 concentrations, which were lower in military police officers than in civilians. In contrast, IL‐2, IL‐17 and IFN‐γ did not demonstrate significant main effects of occupational status in the primary factorial analyses. Thus, rather than indicating a generalized suppression of circulating cytokine responses, the present findings point to a more selective alteration in anti‐inflammatory immune regulation associated with chronic shiftwork and occupational stress exposure.

IL‐10 is a major anti‐inflammatory cytokine involved in limiting excessive inflammatory responses and maintaining immune homeostasis. Reduced circulating IL‐10 concentrations in military police officers might indicate altered anti‐inflammatory regulation and, potentially, diminished capacity for inflammatory resolution (Saraiva et al., 2020). Experimental and clinical studies have shown that chronic sleep disruption and circadian misalignment can influence IL‐10 production through neuroendocrine and autonomic pathways involving glucocorticoid signalling and sympathetic activity (Irwing, 2019). Therefore, the lower IL‐10 concentrations observed in military police officers might reflect stress‐ and circadian‐related alterations in immune homeostasis. Importantly, the occupational effect on IL‐10 remained highly significant after exclusion of extreme observations in the sensitivity analysis, supporting the robustness of this finding.

IFN‐γ plays an important role in Th1‐mediated cellular immunity, antiviral defence, macrophage activation and immune surveillance (Besedovsky et al., 2012; Kak et al., 2018). Although previous studies have suggested that chronic occupational stress, sleep restriction and circadian disruption might affect IFN‐γ production and adaptive immune responsiveness (Heffner et al., 2011; Wright et al., 2015, no significant occupational effect on circulating IFN‐γ was detected in the present cohort. This finding remained unchanged after exclusion of extreme observations, indicating that the absence of an occupational effect was not attributable to the most extreme values. Therefore, our findings do not support a generalized impairment of Th1‐mediated immune responsiveness in these military police officers.

IL‐17 is traditionally recognized as a pro‐inflammatory cytokine but also contributes to mucosal defence and antimicrobial protection (Amatya et al., 2017). Despite evidence that prolonged glucocorticoid exposure, sympathetic activation and chronic stress might influence Th17‐mediated responses (Dhabhar, 2018), IL‐17 concentrations were not significantly affected by occupational status or physical activity in the primary analysis. The absence of a significant occupational effect persisted after exclusion of extreme observations in the sensitivity analysis. Thus, the present data do not provide evidence of a systematic alteration in circulating IL‐17/Th17‐associated responses related to police occupational status.

IL‐2 also showed no significant occupational effect in the primary analysis. Interestingly, an occupational effect emerged after exclusion of extreme observations in the sensitivity analysis (*P* = 0.010, η_p_^2^ = 0.075). Because this association was not observed in the prespecified primary analysis and became evident only after removal of extreme values, it should be regarded as exploratory rather than as a definitive occupational effect. Further studies specifically designed to investigate T‐cell‐related cytokine responses in controlled circadian conditions are required to determine the physiological relevance and reproducibility of this observation (Wright et al., 2015).

Interestingly, physical activity status did not demonstrate significant main effects on any of the evaluated cytokines in the primary analyses. Although exercise is widely recognized as an important modulator of immune function (Nieman and Wentz, 2019), the present findings suggest that habitual physical activity, at the levels observed in this cohort, was not independently associated with circulating IL‐2, IL‐10, IL‐17 or IFN‐γ concentrations. In contrast, the robust occupational effect observed for IL‐10 suggests that chronic shiftwork might be particularly relevant to anti‐inflammatory regulation in this population. This interpretation is consistent with evidence that shiftwork‐related physiological stress can influence immune homeostasis even in physically active individuals (Moreno et al., 2019).

The higher serum 11‐deoxycortisol concentrations observed in military police officers might provide additional evidence of neuroendocrine adaptation associated with chronic occupational and circadian stress. 11‐Deoxycortisol is an immediate precursor in cortisol biosynthesis, and alterations in its circulating concentration might reflect changes in adrenal glucocorticoid steroidogenesis. The hypothalamic–pituitary–adrenal axis is strongly influenced by circadian organization, sleep–wake cycles and stress exposure, and circadian misalignment and sleep disruption have been shown to modify cortisol dynamics and neuroendocrine regulation (Besedovsky et al., 2012; Irwin, 2019; Morris et al., 2012, 2016; Wright et al., 2015). In the present study, occupational status significantly influenced 11‐deoxycortisol concentrations, with higher overall values in military police officers than civilians, whereas physical activity had no significant effect. This pattern might therefore be compatible with altered adrenal steroidogenic regulation associated with chronic shiftwork and repeated occupational stress exposure (Dhabhar, 2018; Moreno et al., 2019; Wright et al., 2015). However, because 11‐deoxycortisol was measured at a single morning time point, and cortisol and ACTH were not assessed simultaneously, the physiological significance of this difference should be interpreted cautiously. The present findings cannot establish increased cortisol production or hypothalamic–pituitary–adrenal axis dysfunction but suggest that adrenal steroidogenesis might represent an additional physiological pathway influenced by chronic occupational circadian disruption.

The present study also demonstrated significant differences in haemoglobin concentrations according to occupational status and occupational status × physical activity interaction. Physically active civilians presented higher haemoglobin concentrations than physically active military police officers. Although the mechanisms underlying this finding remain speculative, chronic sleep restriction and chronic shiftwork have been associated with altered erythropoietic regulation and impaired hormonal control involving cortisol and erythropoietin pathways (Violanti et al., 2012). In addition, chronic low‐grade inflammation might have a negative influence on erythrocyte homeostasis (Weiss et al., 2019).

Platelet counts also demonstrated a significant interaction effect, although pairwise comparisons did not reach significance after Bonferroni adjustment. Platelets are increasingly recognized as important mediators linking inflammation, endothelial dysfunction and cardiovascular risk (Wirth et al., 2017). Previous studies have demonstrated altered platelet activation in individuals exposed to chronic shiftwork (Wright et al., 2015). Although the present study did not directly assess platelet activation markers, the observed interaction effect suggests possible occupational influences on haematological regulation.

Some limitations of the present study should be acknowledged. First, the cross‐sectional design does not allow causal inference regarding the observed associations. Second, although all participants were evaluated within the same standardized morning period (08:00–11:00 h) and military police officers were assessed during an off‐duty/rest period, internal circadian phase was not determined objectively using markers such as dim‐light melatonin onset. Therefore, residual differences in circadian phase between shiftworking police officers and civilians cannot be excluded. Third, daytime sleepiness and physical activity were assessed using the self‐reported Epworth sleepiness scale and IPAQ questionnaires, respectively. Although these are validated instruments, self‐reported measures are susceptible to recall and reporting bias and might not fully capture sleep fragmentation, sleep–wake patterns or the intermittent and variable physical demands associated with police work. The absence of objective assessments, such as actigraphy for sleep–wake patterns and accelerometry for physical activity, limits the characterization of these exposures and might have resulted in underestimation of their magnitude. Future studies combining validated questionnaires with objective monitoring are warranted. In addition, although age did not differ significantly among groups, the relatively broad age range (25–59 years) might have contributed to interindividual variability, and residual age‐related confounding cannot be excluded completely. Several cytokine outcomes also exhibited extreme observations; however, sensitivity analyses excluding objectively defined extreme values confirmed the robust occupational effect on IL‐10 and the absence of significant occupational effects for IL‐17 and IFN‐γ. Finally, the study included only male participants, limiting generalization to female police populations. Nevertheless, important strengths include the factorial design, standardized morning assessments, simultaneous evaluation of cardiovascular autonomic and immune outcomes and inclusion of both occupational and physical activity factors in the analytical model. In addition, HRV was assessed using a single 10 min resting recording. Although this approach permits standardized assessment of short‐term cardiac autonomic modulation, it does not capture the temporal or circadian variation in autonomic activity. Consequently, the HRV findings should be interpreted as differences in resting autonomic modulation rather than as direct evidence of altered circadian rhythmicity. Future studies incorporating repeated assessments across the 24 h cycle or ambulatory 24 h ECG monitoring would allow a more comprehensive evaluation of circadian autonomic regulation in shiftworkers. Habitual dietary intake was not assessed systematically, and although participants were instructed to abstain from caffeine, alcohol and vigorous physical activity for ≥24 h before HRV assessment, residual effects of habitual dietary and stimulant consumption cannot be excluded. Medication use was recorded; however, participants were not specifically excluded or statistically stratified according to all medications capable of influencing autonomic cardiovascular regulation, including β‐blockers. These factors might therefore represent sources of residual confounding. Furthermore, melatonin or other objective markers of endogenous circadian phase were not assessed, precluding direct characterization of circadian phase or melatonin rhythmicity. Finally, the study included only male participants, which limits the generalizability of the findings to female military police officers. Sex‐related differences in autonomic cardiovascular regulation, neuroendocrine stress responses, immune function and physiological responses to circadian disruption might result in different patterns among female officers. In addition, menstrual cycle phase and hormonal status might also influence these responses. Future studies should therefore include both male and female police officers and consider sex‐specific and hormonal factors when examining the physiological consequences of chronic shiftwork. Importantly, the present study was not designed to examine dose–response or time‐of‐day effects of physical activity. Although physical activity was assessed using the IPAQ, detailed exercise volume, intensity and timing were not quantified systematically, precluding dose‐ or time‐dependent analyses. Future studies using objective activity monitoring should investigate exercise dose and timing relative to the occupational duty–recovery cycle in shiftworking police officers. Finally, the cross‐sectional design prevents determination of whether the observed autonomic profile represents adaptive resilience or longer‐term maladaptive autonomic changes; longitudinal studies are required to distinguish these possibilities.

## 5 CONCLUSION

In conclusion, chronic occupational exposure to shiftwork in military police officers was associated with alterations in autonomic cardiovascular regulation, systemic immune regulation and perceived health outcomes. Regular physical activity somewhat improved quality‐of‐life domains; however, several physiological alterations remained predominantly associated with occupational status. The lower circulating IL‐10 concentrations observed in military police officers suggest altered anti‐inflammatory regulation associated with chronic occupational and circadian stress, whereas IL‐2, IL‐17 and IFN‐γ were not significantly affected by occupational status in the primary analyses. These findings reinforce the importance of preventive occupational health strategies targeting circadian health, cardiovascular regulation and immune homeostasis in police populations.

## AUTHOR CONTRIBUTIONS

Hélida C. Aquino‐Santos, Jeanne S. Tavares‐Vasconcelos, Anamei Silva‐Reis, Maysa A. R. Brandão‐Rangel, Rayssa T. Morais‐Felix, Fernando A. Santa‐Rosa, André L. L. Bachi, Viviane Soares and Claudio R. Frison were responsible for investigation; methodology, validation and visualization. Omid Salehian, Rodolfo P. Vieira and Regiane Albertini were responsible for conceptualization, data curation, formal analysis, supervision, validation and writing – original draft. In addition, Rodolfo P. Vieira was responsible for funding acquisition and project administration. All authors approved the final version of the manuscript and agree to be accountable for all aspects of the work in ensuring that questions related to the accuracy or integrity of any part of the work are appropriately investigated and resolved. All persons designated as authors qualify for authorship, and all those who qualify for authorship are listed.

## CONFLICT OF INTEREST

The authors declare no conflict of interest related to this publication.

## GENERATIVE AI STATEMENT

We declare that AI (ChatGPT) was used to correct the manuscript, considering that English is not the native language of all the authors.

## Data Availability

The raw data that support the findings of this study are available upon request to the corresponding author.
